# Evaluation of SARS-CoV-2 viral RNA in fecal samples

**DOI:** 10.1186/s12985-020-01359-1

**Published:** 2020-06-30

**Authors:** Alvaro Mesoraca, Katia Margiotti, Antonella Viola, Antonella Cima, Davide Sparacino, Claudio Giorlandino

**Affiliations:** ALTAMEDICA, Human Genetics laboratories, Altamedica Main Centre, Artemisia SpA, Viale Liegi 45, 00198 Rome, Italy

**Keywords:** Acute respiratory syndrome coronavirus 2 (SARS-CoV-2), SARS-CoV-2 viral RNA, Novel coronavirus disease 19 (COVID-19), Fecal specimens, Respiratory specimens

## Abstract

The need for timely establishment of a complete diagnostic protocol of severe acute respiratory syndrome coronavirus 2 (SARS-CoV-2) is demanded worldwide. We selected 15 positive novel coronavirus disease 19 (COVID-19) patients with mild or no symptom. Initially, fecal samples were negative in the 67% (10/15) of the cases, while 33% (5/10) of the cases were positive. After serial virus RNA testing, 73% (11/15) of the cases resulted positive to fecal specimens. In particular, 15 days after the first positive respiratory specimens test, 6 fecal specimens became positive for SARS-CoV-2 RNA, while 13 respiratory test returned negative result. In conclusion, qRT-PCR assays of fecal specimens, is an important step to control infection, suggesting that samples remained positive for SARS-CoV-2 RNA longer time then respiratory tract samples. Our results enhance the recent knowledge on this emerging infectious disease and offer suggestions for a more complete diagnostic strategy.

## Introduction

On December 31st 2019 China reported the first cases of atypical pneumonia in Wuhan, the capital of Hubei province. The causative virus was found to be a Betacoronavirus, closely related to the severe acute respiratory syndrome coronavirus (SARS-CoV-1) from 2003 and similar to Sarbecoviruses isolated from bats [1, 2]. It was therefore termed SARS-CoV-2 and the disease it causes was named COVID-19 (Corona Virus Disease 2019) [3]. The virus genome and early epidemiological and clinical features of the infection in adults have been reported [4–6]. The infection is estimated to have about 5 days of incubation period and commonly causes fever, cough, myalgia and pneumonia in patients [4]. Several real time RT-PCR assays protocols for detection of SARS-CoV-2 RNA have been developed and approved from Centers for Disease Control and Prevention Nucleic acid in US and are now widely employed to diagnose COVID-19 disease [7, 8]. Current scientific literature has shown that viral RNA measurements from fecal specimens might be last longer than that from respiratory tract [9, 10]. Herein, we selected fifteen positive patients for SARS-CoV-2 RNA in their respiratory tract, and we followed the viral shedding from respiratory and gastrointestinal tracts by a series of respiratory and fecal specimens test by real time RT-PCR assays. Has been reported that the duration of viral shedding in fecal specimens, can be detected for nearly 5 weeks after that the patients’ respiratory samples tested negative for SARS-CoV-2 RNA [9]. Moreover, recent meta-analysis on 4243 COVID-19 patients reported that stool samples from 48.1% of patients tested positive for virus RNA, among these patients 70.3% tested positive for RNA virus even after respiratory specimens tested negative [11]. The aim of this study was to evaluate SARS-CoV-2 virus fecal shedding and duration in comparison with SARS-CoV-2 virus in the respiratory tract. Determine whether a virus is still present in the gastrointestinal tract of the patients several days after respiratory tract samples became negative, is important in order to understand and define a complete diagnostic strategy for SARS-CoV-2 viral infection.

## Results and discussion

Between March 4 and April 20, 2020, we collected every 5 days respiratory and fecal specimens from fifteen COVID-19 patients. All 15 samples tested positive in their respiratory swab in the first 10 days, when all internal controls exhibit the expected performance, a specimen is considered positive if two out of three SARS-CoV-2 target genes (gene E, gene N, gene ORF1ab) showed a cycle threshold (CT) values ≤41, as indicated on manufactural guidelines (Novel Coronavirus (2019-nCoV) Real Time Multiplex RT-PCR kit, LifeRiver Ltd). Among this 15 samples 67% (10/15) tested negative in their fecal specimens, and 33% (5/10) tested positive in their fecal specimens as well (Table 1). Of the 10 patients with fecal sample negative in the first 10 days, 6 samples became positive 2 weeks after their first positive respiratory tract test as shown in Table 1. Out of this 11 fecal specimens positive for SARS-CoV-2 RNA virus, 6 samples remained positive until the end of the sampling for additional 15 days as shown in Table 1. Interesting, 4 patients had fecal samples positive test 25 days more than their respiratory tract test (Table 1). Our data are in accordance with previous study were has been shown that rectal swabs can become and remain positive for longer time after the respiratory specimens testing had become negative [9, 10]. Even though patients had mild or non-symptoms, is well none that asymptomatic transmission is possible, and potential fecal-oral transmission must to be considered to reduce the risk of transmission. Respiratory transmission is still the primary route for SARS-CoV-2 infection and further research on this field must be done in order to understand SARS-CoV-2 virus better and implement the diagnostic strategy. Our preliminary data support the idea that negative fecal result, might be a necessary criterion in addition to the actual two consecutive negative testing in respiratory samples, before considering the patients completely recovered and no more infective.

**Table 1:** Patients respiratory and fecal specimens results for SARS-CoV-2 RNA test

Case	Gender	Symptoms	Specimens	1-10 days	15–25 days	30–40 days
1	M	Fever	RT	Positive	Positive	Negative
			FS	Negative	Positive	Positive
2	M	none	RT	Positive	Negative	Negative
			FS	Negative	Negative	Negative
3	M	Fever	RT	Positive	Negative	Negative
			FS	Negative	Negative	Negative
4	M	none	RT	Positive	Negative	Negative
			FS	Negative	Positive	Positive
5	M	Fever	RT	Positive	Negative	Negative
			FS	Positive	Positive	Negative
6	M	none	RT	Positive	Negative	Negative
			FS	Negative	Positive	Positive
7	M	none	RT	Positive	Negative	Negative
			FS	Negative	Positive	Negative
8	M	Fever	RT	Positive	Negative	Negative
			FS	Negative	Negative	Negative
9	M	none	RT	Positive	Positive	Positive
			FS	Positive	Positive	Negative
10	F	Fever	RT	Positive	Negative	Negative
			FS	Negative	Positive	Positive
11	F	none	RT	Positive	Negative	Negative
			FS	Positive	Positive	Negative
12	F	none	RT	Positive	Negative	Negative
			FS	Positive	Positive	Negative
13	F	none	RT	Positive	Negative	Negative
			FS	Negative	Negative	Negative
14	F	none	RT	Positive	Negative	Negative
			FS	Positive	Positive	Negative
15	F	Fever	RT	Positive	Negative	Negative
			FS	Negative	Positive	Positive

*RT* Respiratory tract, *FS* Fecal specimens, *F* Female, *M* Male

## Methods

A total of fifteen patients with mild or no symptoms were selected for this study. This case series was approved by the institutional Ethics Committee of Artemisia SpA. Fifteen patients with respiratory specimens positive for SARS-CoV-2 RNA virus from March 4, to April 20, 2020 were enrolled. Oral consent was obtained from patients. The respiratory and fecal specimens were collected every 5 days from March 4, 2020, until final date of the study. The diagnosis by real-time RT-PCR were performed in our institute, extraction of nucleic acids from the respiratory and fecal samples was performed with the commercialized nucleic acid extraction kits (QIAamp Viral RNA Mini Kit, Catalog #: 52904, QIAGEN). The Real-time RT-PCR was done by using the Novel Coronavirus (2019-nCoV) Real Time Multiplex RT-PCR kit from LifeRiver Ltd., and iQ5 real-time PCR detection system (Bio-Rad, Hercules, CA, USA) following the manufacture instruction. The kit measures simultaneously 3 target genes: SARS-CoV-2 gene E, gene N, gene ORF1ab thus complying with international validated testing protocols (Novel Coronavirus (2019-nCoV) Real Time Multiplex RT-PCR kit, LifeRiver Ltd).

## Conclusion

A fecal SARS-CoV-2 RNA virus test using real-time RT-PCR techniques was used to evaluate the virus shedding 2 weeks after a positive respiratory tract swab. Results from this study demonstrated that patients had fecal samples positive test 25 days more than their respiratory tract test. Some limitations refer to the number of samples analysed, more samples will better define the virus shedding in feces and the utility to recommend routine fecal specimens tasting. In conclusion, fecal specimens has great potential benefit for more accurate screening of SARS-CoV-2 infections, and it has already generated vast interest in the medical community.

## Data Availability

Not applicable.

## Abbreviations

COVID-19: Corona Virus Disease 2019
SARS-CoV-2: Acute respiratory syndrome coronavirus 2
